# Bacteriology in uncomplicated urinary tract infections in Norwegian general practice from 2001–2015

**DOI:** 10.3399/bjgpopen17X101145

**Published:** 2017-10-04

**Authors:** Marianne Bollestad, Ingvild Vik, Nils Grude, Hege Salvesen Blix, Hanne Brekke, Morten Lindbaek

**Affiliations:** 1 Infectious Diseases Resident & PhD Student, Department of General Practice, Antibiotic Centre of Primary Care, Institute of Health and Society, University of Oslo, Oslo, Norway; 2 Infectious Diseases Resident & PhD Student, Department of Emergency General Practice, Oslo Accident and Emergency Outpatient Clinic, City of Oslo Health Agency, Oslo, Norway; 3 Infectious Diseases Resident & PhD Student, Division of Medicine, Stavanger University Hospital, Stavanger, Norway; 4 GP & PhD Student, Department of General Practice, Antibiotic Centre of Primary Care, Institute of Health and Society, University of Oslo, Oslo, Norway; 5 GP & PhD Student, Department of Emergency General Practice, Oslo Accident and Emergency Outpatient Clinic, City of Oslo Health Agency, Oslo, Norway; 6 Consultant Microbiologist & Researcher, Department of General Practice, Antibiotic Centre of Primary Care, Institute of Health and Society, University of Oslo, Oslo, Norway; 7 Consultant Microbiologist & Researcher, Department of Medical Microbiology, Vestfold Hospital Trust, Toensberg, Norway; 8 Specialist in Hospital Pharmacy, Norwegian Institute of Public Health, Oslo, Norway; 9 Infectious Diseases Resident, Department of Medical Microbiology, Oslo University Hospital, Oslo, Norway; 10 Professor, Department of General Practice, Antibiotic Centre of Primary Care, Institute of Health and Society, University of Oslo, Oslo, Norway

**Keywords:** primary health care, urinary tract infection, bacteriuria, female urogenital diseases, anti-bacterial agents, drug resistance

## Abstract

**Background:**

Uncomplicated urinary tract infections in women are common, and urine samples from these patients are not routinely cultured. Empirical treatment is based on knowledge of resistance patterns for common uropathogens.

**Aim:**

To evaluate the bacteriological findings and resistance patterns in urine samples from women with uncomplicated urinary tract infections, and to assess the relationship between antimicrobial use and resistance patterns from 2000–2015 in Norway.

**Method:**

Bacteriology and resistance patterns were compared in 184 urine cultures from 2001, 406 urine cultures from 2010–2011 and 259 urine cultures from 2013–2015. Antibiotic use data from 2000–2015 were obtained from national databases.

**Results:**

*Escherichia coli* (*E. coli*) was the main bacterial agent in 80% of the cultures. *Staphylococcus saprophyticus* (*Staph. saprophyticus*) represented 6–17%. For *E. coli*, resistance to mecillinam showed some variation but remained below 9%. There was negligible resistance to nitrofurantoin. Resistance to trimethoprim seemed to stabilise over the last 5 years at around 20%. Amoxicillin resistance had some variations, but remained stable around 30%. There was a steady rise in total consumption of selected antibiotics commonly used to treat urinary tract infections for the period 2000–2015.

**Conclusion:**

Mecillinam and nitrofurantoin are both excellent first choices for empirical treatment of uncomplicated urinary tract infections. This study suggests that increasing resistance to trimethoprim challenges the rationale for its use as a first-line agent.

## How this fits in

Empirical treatment for acute uncomplicated UTI is based on current resistance patterns and national guidelines. This study evaluated antibiotic use and bacteriological findings in uncomplicated UTIs in Norway from 2001–2015. This study found stable and high susceptibility to mecillinam and nitrofurantoin for *E. coli*. This study suggests that increasing resistance to trimethoprim challenges the rationale for its use as a first-line agent.

## Introduction

Uncomplicated UTI is the most common infection in women presenting to primary health care.^1^ Most women with symptoms of a UTI consult a doctor and are prescribed antibiotics.^2^ Empirical treatment for uncomplicated UTI is based on current resistance patterns and national guidelines.^3^ Treatment choices made in primary health care are important as they account for 85% of the total antibiotic use in Norway.^4^

*E. coli* is the most common pathogen isolated in community-acquired UTI.^5,6^ Other pathogens commonly identified are *(Staph. saprophyticus, Klebsiella species (spp.), Enterococcus spp., Enterobacter spp.* and *Proteus mirabilis*.^7^

Current Norwegian guidelines recommend mecillinam, nitrofurantoin, and trimethoprim as empirical treatment options for uncomplicated UTI.^3^ Previous studies have sought to determine resistance rates to empirical treatment regimes.^8,9^

Data from Belgium have suggested that species distribution is relatively stable, and that sensitivity to fosfomycin and nitrofurantoin remain at nearly 100%.^10,11^ However, international data show increasing antimicrobial resistance to several recommended empirical treatment regimes.^12–14^ Interestingly, Poland recently reported high overall resistance rates of the common uropathogens to first-line treatment regimes, including fosfomycin and nitrofurantoin.^12^

Current guidelines suggest the diagnosis may be given based on symptoms alone.^3,15^ Since 2000 the Norwegian guidelines have recommended not to routinely culture urine samples from patients with acute uncomplicated UTI. This may lead to potential changes in resistance patterns going undetected and an overestimation of resistance rates.^16–18^

The aims of the study were:

- to evaluate the bacteriological findings and resistance patterns in urine samples from women with acute uncomplicated UTI in three cohorts in the time period 2001–2015 in Norway; and
- to assess the relationship between the use of antimicrobial agents in the treatment of UTI and resistance patterns in the time period 2000–2015 in Norway.

### Method

The bacteriology and resistance patterns were compared in urine cultures collected from women with uncomplicated UTIs presenting in a general practice setting in three different time periods in Norway. Inclusion and exclusion criteria for the different studies have been published.^8,19–20^ The material consisted of 184 urine cultures from 2001, 406 urine cultures from 2010–2011, and 259 urine cultures from 2013–2015.

The first study was performed in the county of Telemark in 2001. This study enrolled arbitrarily selected women presenting to the GP with symptoms of an uncomplicated UTI for which they received antibiotics.^8^ Fresh midstream urine samples were sent to the local microbiology department in sterile containers with 1.6% boric acid. Significant bacteriuria was defined as pure or dominant growth of ≥10^4^ colony-forming units per millilitre (cfu/mL) for all pathogens. Antimicrobial susceptibility breakpoints were set according to the Norwegian Working Group on Antibiotics.^21^

The next two studies were performed at Oslo Accident and Emergency Outpatient Clinic (OAEOC), Department of Emergency General Practice. Sequential consulting women were enrolled in the time periods September 2010–November 2011 and April 2013–December 2015.

In the 2010–2011 study, inclusion criteria were determined by a diagnostic algorithm based on established symptoms and risk factors for complicated UTI. This study was conducted to validate a specific diagnostic algorithm and the results have been published.^19^

In the 2013–2015 study the same inclusion criteria were used to identify women with uncomplicated UTI. The data presented here are baseline data from the main centre of an ongoing multicentre randomised controlled trial (NCT01849926) to assess ibuprofen versus mecillinam in the treatment of uncomplicated UTI.^20^

For the two latter studies a fresh midstream urine sample was sent to the Department of Microbiology at Oslo University Hospital, Ullevål, in sterile containers with 1.6% boric acid. The uropathogens were quantified in cfu/mL. Significant bacteriuria was defined according to current European guidelines as ≥10^3^/mL for primary pathogens, ≥10^4^/mL for secondary pathogens, and ≥10^5^/mL for doubtful pathogens.^22^ Clinical breakpoints were taken from the European Committee on Antimicrobial Susceptibility Testing, which have remained unchanged since 2010.^23^

Data on antibiotic use were collected from two nationwide databases; the Norwegian drug wholesale statistics database and the Norwegian prescription database (NorPD).^24^ The wholesale database logs sales of all medicines in Norway. NorPD contains a complete listing of all prescription drugs dispensed by pharmacies in Norway since 2004.

National resistance patterns were collected from The Norwegian Organization for Surveillance of Antimicrobial Resistance (NORM). From 2000 onwards, NORM has provided annual reports on the national usage of antimicrobial agents and the occurrence of resistance in Norway.^4^ Data on resistance were compared to the total use (wholesales statistics) of selected antibiotics.

The results in this study represent a comparison done retrospectively and a power calculation was not performed. IBM SPSS statistics (version 23.0) was used for descriptive analyses of data. Comparison of aggregated data was done using a χ^2^ calculator with a significance level of 0.05.^22^

### Results

The three study populations all represented adult women, aged 15–65 years, with uncomplicated UTIs. There was a difference in age distribution between the first population and the two latter populations, with the first group representing an older cohort. The mean age in the two groups from the OAEOC was 27.1 years, whereas the first group had a mean age of 38.3 years. There was no statistically significant difference in age between the two groups from the OAEOC, but when each of the OAEOC groups were compared to the first group, the differences in age distribution were statistically significant with a *P*-value varying from *P *= 0.03 to *P*<0.01.

Significant bacterial growth was found in 57–74% of all urine samples cultured. The main bacteriological agent in all three study populations was *E. coli,* ranging from 78% to 82%. The second most common isolate was *Staph. saprophyticus,* ranging from 6% to 17% (Table 1).

**Table 1.** Bacterial isolates from the three study cohorts

Microbe	2001 *n* (%)	2010–2011 *n* (%)	2013–2015 *n* (%)
*E. coli*	112 (82.4)^a^	180 (78.3)^a^	129 (81.6)^a^
*Staph. saprophyticus*	8 (5.9)^a^	38 (16.5)^a^	22 (13.9)^a^
*Enterobacter spp*.	0^a^	1 (0.4)^a^	4 (2.5)^a^
*Enterococcus faecalis*	3 (2.2)^a^	2 (0.9)^a^	1 (0.6)^a^
*Klebsiella spp.*	6 (4.4)^a^	5 (2.2)^a^	2 (1.3)^a^
*Proteus spp.*	5 (3.7)^a^	4 (1.7)^a^	0^a^
*Staph. aureus*	2 (1.5)^a^	0^a^	0^a^
No significant growth	48 (26.1)^b^	176 (43.3)^b^	101 (39.0)^b^
Total number of cultures with significant bacteriuria	136 (73.9)^b^	230 (56.7)^b^	158 (61.0)°^b^
Total number of cultures	184	406	259

^a^% of total number of cultures with significant bacteriuria. ^b^% of total number of cultures.

For trimethoprim there was a substantial increase in resistance from 2001 to 2010–2011, then a slight decrease from 2010–2011 to 2013–2015. There was a decrease in resistance to mecillinam in *E.coli* from 2001 to 2010–2011, followed by an increase from 2010–2011 to 2013–2015. There was an increase in resistance to amoxicillin for *Staph. saprophyticus* isolates from 2010–2011 to 2013–2015. There was no increase in resistance to nitrofurantoin for neither *E. coli* or *Staph. saprophyticus.* None of the changes in resistance rates were statistically significant (Table 2).

**Table 2.** Resistance to antibiotics commonly used to treat UTIs

Microbe	Antibiotic	2001 *n* (%) [95 % CI]	2010–2011 *n* (%) [95 % CI]	2013–2015 *n* (%) [95 % CI]
*E. coli*	Mecillinam	7 (6.3) [1.8 to 10.8]	4 (2.2) [0.1 to 4.3]	11 (8.5) [3.7 to 13.3]
	Nitrofurantoin	3 (2.7) [0.0 to 5.7]	0	0
	Trimethoprim	13 (11.6) [5.7 to 17.5]	38 (21.1) [15.1 to 27.1]	27 (20.9) [13.9 to 27.9]
	Co-trimoxazole	NA	39 (21.7) [15.7 to 27.7]	24 (18.6) [11.9 to 25.3]
	Sulphonamide	21 (18.8) [11.6 to 26.0]	NA	NA
	Amoxicillin	31 (27.7) [19.4 to 36.0]	63 (35.0) [28.0 to 42.0]	38 (29.5) [21.6 to 37.4]
	Ciprofloxacin	NA	NA	NA
*Staph. saprophyticus*	Mecillinam	NA	NA	NA
	Nitrofurantoin	0	0	0
	Trimethoprim	0	0	1 (4.5) [–4.2 to 13.2]
	Co-trimoxazole	NA	0	0
	Sulphonamide	0	NA	NA
	Amoxicillin	NA	4 (10.5) [0.8 to 20.2]	4 (18.2) [2.1 to 34.3]
	Ciprofloxacin	NA	NA	NA

NA = not applicable, isolate was not tested for this antibiotic.

**Figure 1:**
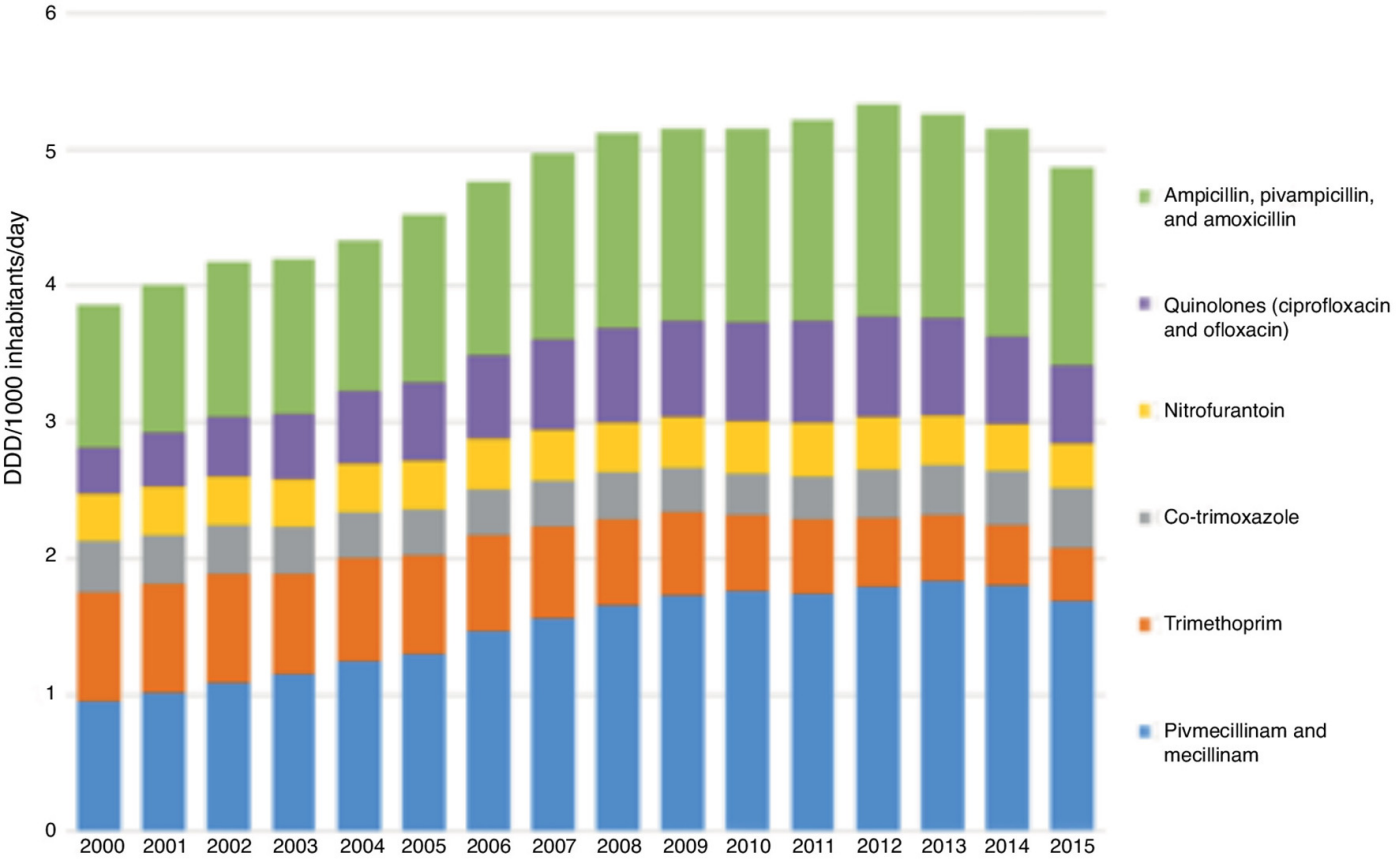
Total use of selected antibiotics commonly used to treat urinary tract infections in Norway (wholesale statistics).^a^In 2000-2002, nalidixic acid and sulfathiazole were also used in Norway, however these two represented less than 0.3% of total use of antibiotics with the indication urinary tract.

**Figure 2:**
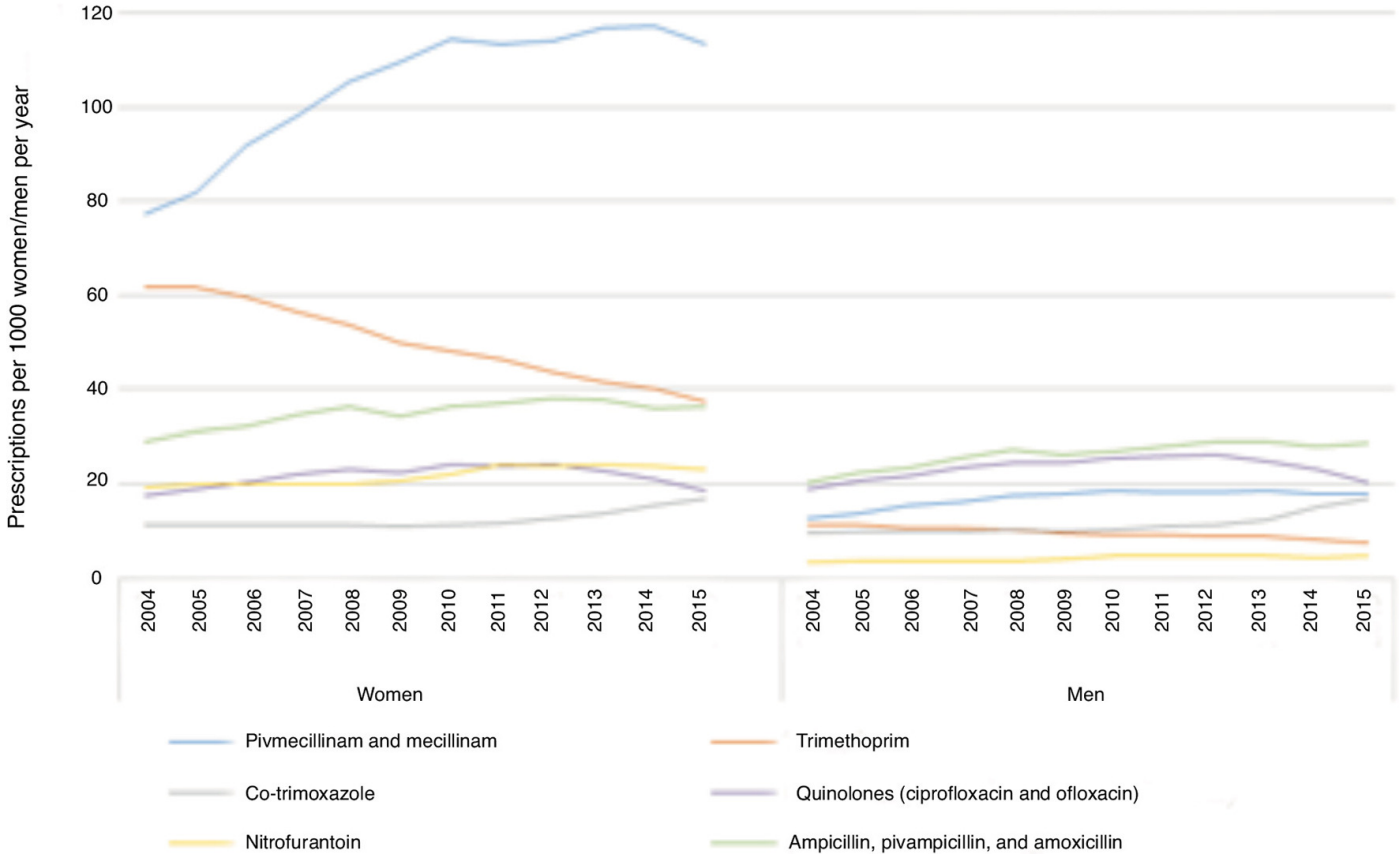
Distribution of use measured as number of prescriptions for selected antibiotics per 1000 women and men >20 years of age.

Wholesale statistics for commonly used antibiotics showed a steady rise in total consumption from 2000 until 2012. However, since 2012, there has been an overall reduction in antibiotic use (Figure 1). Data for the first choice antibiotics in the treatment of uncomplicated UTIs showed an increase in mecillinam consumption and a decrease for trimethoprim, while the use of nitrofurantoin and co-trimoxazole remained stable. Other treatment options for UTIs include amoxicillin/ampicillin and the quinolones ciprofloxacin and ofloxacin. For these drugs, there was a clear rise in consumption and a rise in resistance rates. However, the use has slightly decreased from 2012 onwards. Women used more antibiotics than men and the patterns of use were different. Women were more frequently prescribed mecillinam, while quinolones were more often prescribed for men (Figure 2).

**Figure 3:**
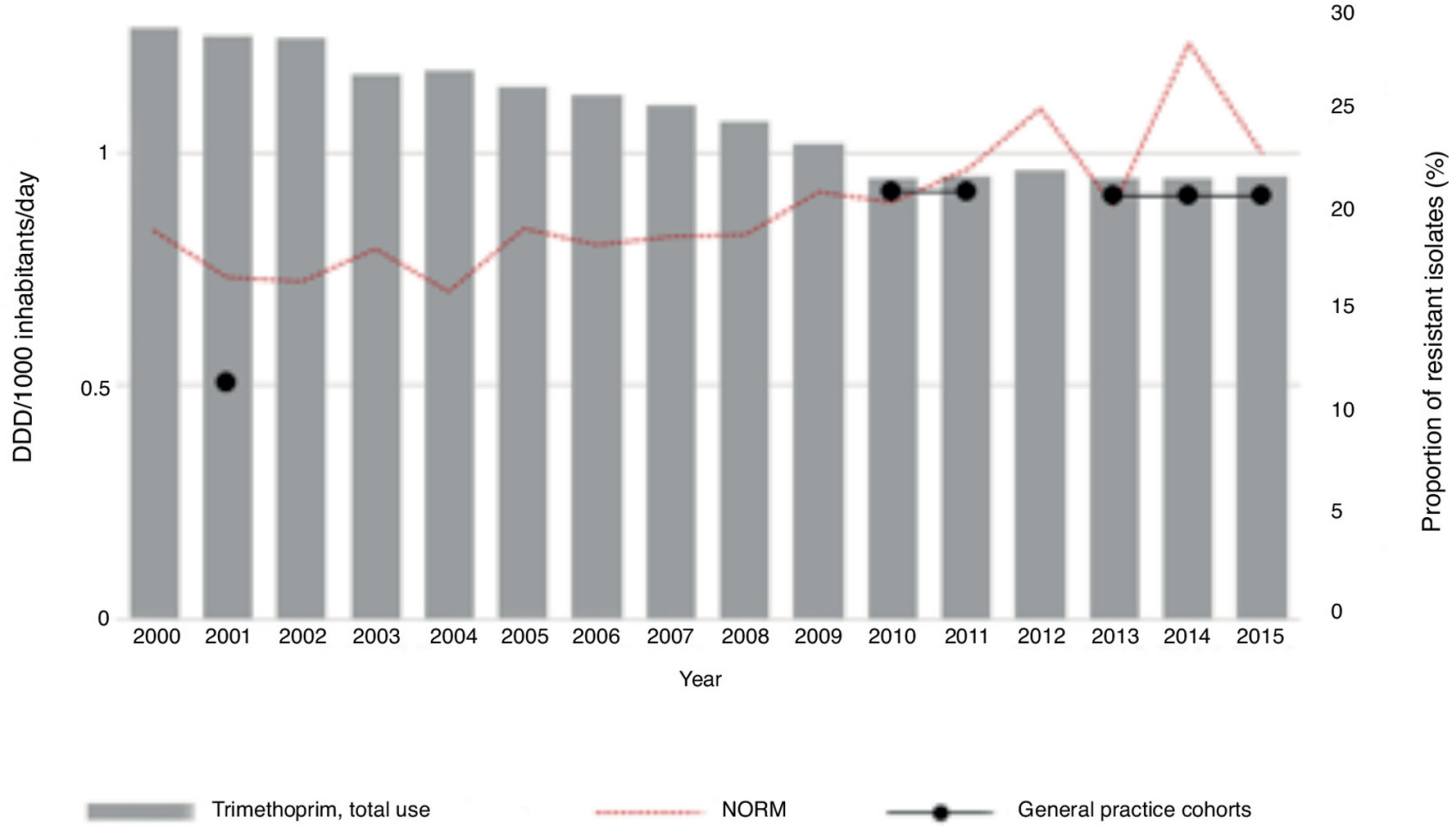
Total use of trimethoprim (wholesale statistics) and prevalence of resistant strains of *E.coli* isolates in urinary tract isolates from the national register (NORM) and the three different general practice cohorts.

**Figure 4:**
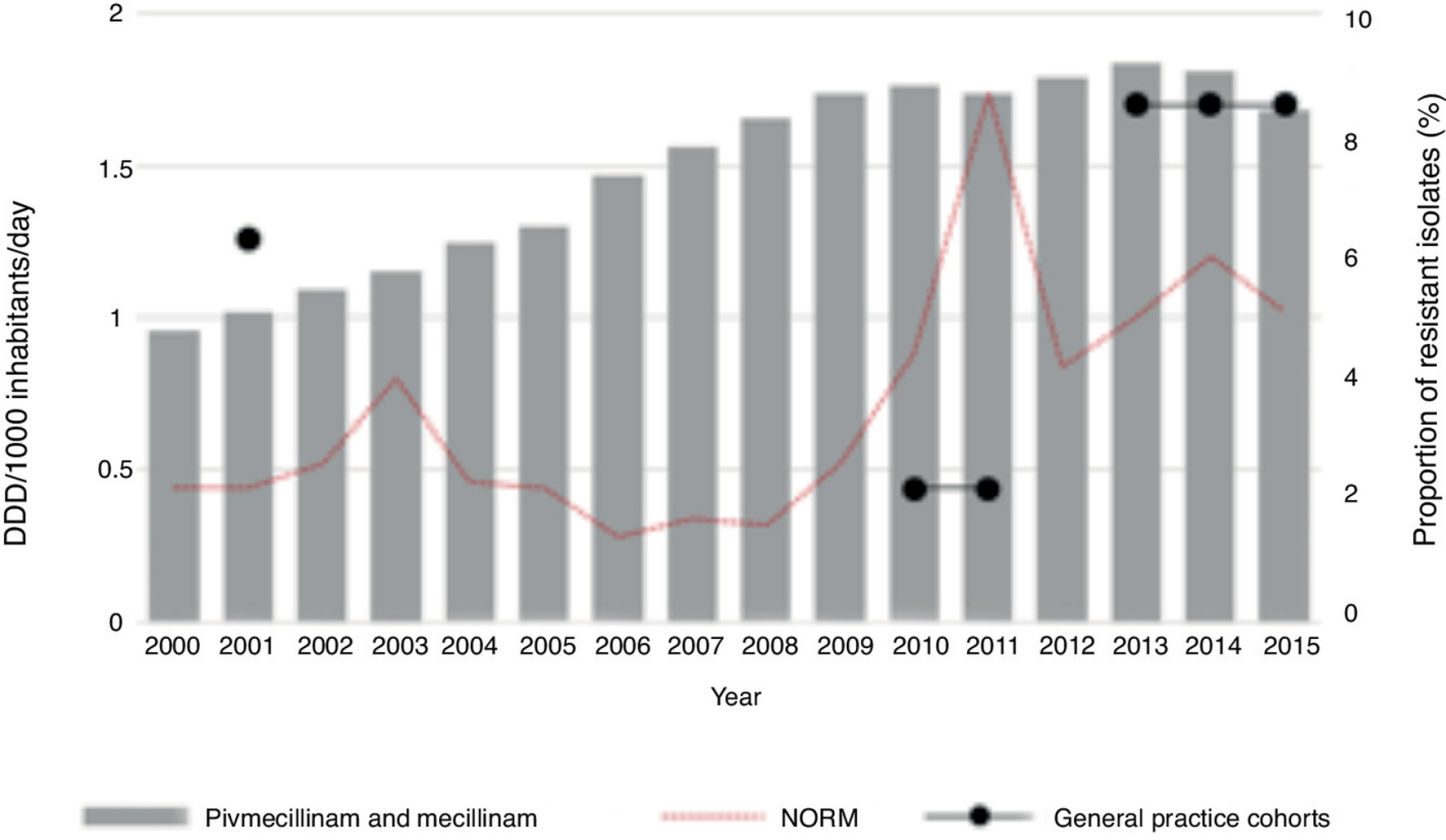
Total use of pivmecillinam and mecillinam (wholesale statistics) and prevalence of resistant strains of *E.coli* isolates in urinary tract isolates from the national register (NORM) and the three different general practice cohorts.

This study looked specifically at the relationship between consumption and *E. coli* resistance rates, both in these and national data, for the two most commonly used antibiotics, trimethoprim and mecillinam. For trimethoprim, there was a reduction in use over the last 15 years. Trimethoprim resistance for *E. coli*, however, slightly increased (Figure 3). For mecillinam there was a clear rise in consumption during the study period, with a minimal decrease over the last 2 years. The resistance rates for *E. coli* showed some variation, but the overall trend was a slight increase in mecillinam resistance (Figure 4).

## Discussion

### Summary

E. *coli* was the dominant pathogen in all three populations. The prevalence of *Staph. saprophyticus* was higher in the 2010–2011 and 2013–2015 cohorts, which was expected as these two groups consisted of younger women.^25–26^

This analysis showed that both *E. coli* and *Staph. saprophyticus* have been fully sensitive to nitrofurantoin over the last 5 years. Use of nitrofurantoin has remained stable and low during this 15-year period. National numbers showed resistance rates of 1% for all urinary isolates of *E. coli* towards nitrofurantoin in 2015.^4^

Trimethoprim resistance increased from 2001 to 2011. However, during the last 5 years this trend has stabilised. These numbers were slightly lower than the national numbers, but the differences were marginal: 21% versus 23% in 2015 (Figure 3). Although the total use of trimethoprim has decreased, the resistance level has increased in the last 15 years. Even though these numbers are lower than the national numbers, the resistance rates exceed 20%. Norwegian guidelines suggest that antimicrobial agents should not be used empirically if resistance rates exceed 20%.^3^

There was some variation in the level of resistance to mecillinam in *E. coli*. In the current comparison there was a 'dip' in resistance for 2010–2011. This does not correspond with the findings in the national report, which showed a peak in the same period. In the study populations from 2001 and 2013–2015, there were higher resistance rates than the national rates. A possible explanation for this is that resistance to mecillinam in *E. coli* is difficult to measure and might have some geographical variations. These numbers differed somewhat from the national numbers, but both suggest quite stable and low levels of resistance. Despite a substantial rise in the use of mecillinam in Norway, there has not been a corresponding increase in the level of resistance to mecillinam for *E. coli*. This indicates that mecillinam has a low potential for inducing resistance.

For *E. coli* the resistance to amoxicillin also showed some variation, but remained high around 30%. Resistance levels were slightly lower in the current analysis than the national numbers, but again the observed differences were minimal: 30% versus 34% in 2015.

For amoxicillin, there was a marked increase in resistance in *Staph. saprophyticus*. Antibiotic resistance in *Staph. saprophyticus* is infrequent,^27^ and resistance patterns for this bacteria are not included in national surveillance data. *Staph. saprophyticus* is not considered to be sensitive to mecillinam and susceptibility testing is therefore not routinely performed.^6^

### Strengths and limitations

This analysis has three large general practice cohorts presenting with uncomplicated UTI. In Norway, urine from uncomplicated UTIs has not been routinely cultured in the last 20 years. This study evaluated if there has been a change in the distribution of bacterial isolates and resistance patterns in this population. Looking specifically at this group of patients is important as the national surveillance data represent a diverse population with more complicated infections. A 15-year comparison is a long period and provides confidence that this study's conclusions about the development of antibiotic resistance are accurate.

This study compared cohorts from Oslo and Telemark, which represents two of the 19 counties in Norway. Oslo County consist of the city of Oslo and surrounding suburbs. Telemark County consists of several municipalities and a few cities. Looking at the three cohorts together, there are data representing both an urban and a more rural population, reflecting the general Norwegian population. A limitation is that the two latter populations are similar and represent a younger population. The first dataset are from a more diverse population and this can cause some uncertainty when comparing the three groups. Other complicating factors are different procedures in the microbiological laboratories and changes in breakpoints in susceptibility testing. A weakness of this observational study is that a power calculation was not performed with regards to changes in resistance rates. Consequently, interpretation of significance levels is uncertain. All patients had to consent to participation, which might have resulted in selection bias.

### Comparison with existing literature

Antimicrobial resistance is an international concern, and efforts are being made in many countries to achieve a better overview of consumption of antibiotics and national resistance rates.^28^ Strategies to reduce antibiotic consumption include efforts to identify women with UTI who recover without antibiotics.^29^ The topic of dusting off 'old' antimicrobials to treat UTIs has been raised.^30^ Several European studies suggest that there is little resistance to mecillinam, fosfomycin, and nitrofurantoin, which the current study supports.^9–13,27^ These three antibiotics all seem to be good first choice treatment options for many European countries.

Mecillinam is mainly used in the Scandinavian countries, but it should receive more attention in other European countries. *Staph. saprophyticus* is considered to be intrinsically resistant to mecillinam, and extended spectrum beta-lactamase producing uropathogens are by definition resistant to mecillinam treatment, however smaller clinical studies have shown varying degrees of treatment effect.^6,31–32^ Fosfomycin is widely used in some countries, for example Germany and Spain, but in Norway it is not marketed or readily available. According to data provided by NorPD, only 28 packages were prescribed to 19 patients in 2015. There is a rationale for Norway and the other Nordic countries to consider including fosfomycin as a first-line antibiotic treatment for uncomplicated UTI, replacing trimethoprim which has a resistance level of >20% in most European countries. Studies have shown high susceptibility rates for *Staph. saprophyticus* to fosfomycin.^33–34^ Common European guidelines for the treatment of uncomplicated UTI might help to ensure good antibiotic stewardship.

The current analysis and other studies show that national surveillance numbers for resistance are somewhat higher than in urine cultured from patients with uncomplicated UTI.^11,35–36^ *Staph. saprophyticus* has not been taken into account in the national surveillance of resistant microbes as it has been known to have very little resistance. In this comparison there was an increase in resistance to both amoxicillin and trimethoprim, and even though these numbers are small, they warrant increased monitoring. To keep track of the actual distribution of bacterial isolates and level of resistance it is important to keep a sentinel surveillance of bacterial agents causing uncomplicated UTIs. This is in line with what researchers from other countries have suggested.^11,36^

### Implications for practice

Mecillinam and nitrofurantoin are both excellent first choices for empirical treatment of uncomplicated UTIs. This study suggests that increasing resistance to trimethoprim challenges the rationale for its use as a first-line agent. Norway might consider including fosfomycin as a first choice antibiotic for the treatment of uncomplicated UTI. 

Sentinel surveillance of bacterial isolates from uncomplicated UTIs is necessary to ensure effective empirical treatment options.
